# MyD88 and TRIF mediate divergent inflammatory and regenerative responses to skeletal muscle ischemia

**DOI:** 10.14814/phy2.12006

**Published:** 2014-05-20

**Authors:** Ulka Sachdev, Xiangdong Cui, Jia Xu, Jun Xu, Edith Tzeng

**Affiliations:** 1 University of Pittsburgh Medical Center, Pittsburgh, Pennsylvania; 2 Department of Surgery, VA Pittsburgh Health System, Pittsburgh, Pennsylvania

**Keywords:** Innate immunity, muscle ischemia, PAD, Toll‐like receptors

## Abstract

We have previously shown that MyD88 KO mice appear protected from ischemic muscle injury while TRIF KO mice exhibit sustained necrosis after femoral artery ligation (FAL). However, our previous data did not differentiate whether the protective effect of absent MyD88 signaling was secondary to attenuated injury after FAL or quicker recovery from the insult. The purpose of this study was to delineate these different possibilities. On the basis of previous findings, we hypothesized that MyD88 signaling promotes enhanced inflammation while TRIF mediates regeneration after skeletal muscle ischemia. Our results show that after FAL, both MyD88 KO mice and TRIF KO mice have evidence of ischemia, as do their control counterparts. However, MyD88 KO mice had lower levels of serum IL‐6 24 h after FAL, while TRIF KO mice demonstrated sustained serum IL‐6 up to 1 week after injury. Additionally, MyD88 KO mice had higher nuclear content and larger myofibers than control animals 1 week after injury. IL‐6 is known to have differential effects in myoblast function, and can inhibit proliferation and differentiation. In tibialis anterior muscle harvested from injured animals, IL‐6 levels were higher and the proliferative marker MyoD was lower in TRIF KO mice by PCR. Furthermore, expression of MyD88 appeared to be higher in skeletal muscle of TRIF KO mice. In vitro, we showed that myoblast differentiation and proliferation were attenuated in response to IL‐6 treatment giving credence to the finding that low IL‐6 in MyD88 KO mice may be responsible for larger myocyte sizes 1 week after FAL. We conclude that MyD88 and TRIF work in concert to mediate a balanced response to ischemic injury.

## Abstract (precis)

We describe opposing roles of MyD88 and TRIF, both downstream signaling molecules of TLR4, in the inflammatory and regenerative processes that follow limb ischemia. MyD88 appears to mediate inflammation, while TRIF appears to be required for modulation of MyD88 activity and promoting regeneration. Absence of MyD88 may ultimately have a protective effect in muscle recovery after ischemic injury.

## Introduction

Peripheral arterial disease affects more than five million adults in the United States with a fair number progressing to major amputation when revascularization is unsuccessful (Selvin and Erlinger 2004; Marston et al. 2006). Limb loss is associated with significant morbidity and mortality with 6–12% of these patients dying within 30 days of amputation (Nelson et al. 2012). Thus, a major focus has been placed on deciphering the events that mediate endogenous revascularization so that these mechanisms can be exploited to enhance angiogenesis for limb salvage (Messina et al. 2002; Powell et al. 2004). Proangiogenic approaches that have been evaluated include growth factors, gene transfer, and stem cell therapy with limited success (Masaki et al. 2002; Powell et al. 2004). For instance, VEGF therapy resulted in the formation of blood vessels that were leaky, leading to significant edema (Baumgartner et al. 1998; Rajagopalan et al. 2003). Thus, continued research is required to improve the therapies for patients with critical limb ischemia.

Toll‐like receptors (TLRs), which are critical in mediating inflammatory responses to both endogenous and exogenous pathogens, are key sensors of tissue damage such as that which occurs in the setting of ischemia (Levy et al. 2007). The nuclear protein high‐mobility group box‐1 (HMGB1) is a damage‐associated molecular pattern (DAMP) that is mobilized and released following tissue ischemia and interacts with these TLRs to mediate downstream responses (Lotze and Tracey 2005). While much attention has been on the role of TLRs and DAMPS in inflammation, we and others have focused on evaluating TLR signaling in the regenerative processes. We have previously reported that hindlimb ischemia is tolerated well in C57B6 mice lacking TLR4 while those lacking TLR2 developed marked muscle necrosis with poor regeneration and impaired angiogenesis (Sachdev et al. 2012).

Toll‐like receptors signal through the cytosolic adaptor proteins myeloid differentiation primary response gene 88 (MyD88) and TIR domain containing adapter‐inducing interferon *β* (TRIF). MyD88 signals downstream of TLR2, TLR4, and TLR9 while TRIF mediates TLR3 and TLR4 signal transduction (Baumgartner et al. 1998; Mitchell et al. 2007). We have previously demonstrated that MyD88 knockout (MyD88 KO) mice tolerate hindlimb ischemia very well, showing little evidence of muscle injury (i.e., fat replacement, actively regenerating myocytes) 2 weeks after femoral artery ligation. In stark contrast, TRIF KO mice exhibit pronounced tissue necrosis and inflammatory cell infiltration (Sachdev et al. 2012). These differences occur despite similar levels of perfusion recovery between these strains of mice as well as control animals (Sachdev et al. 2012). However, it is not clear whether the reason for these differences is due to an attenuated response to the ischemic injury or a faster recovery in MyD88 KO mice. Furthermore, we did not describe the potential mechanisms by which TRIF and MyD88 may be promoting the different phenotypes that were observed. On the basis of our previous findings, we hypothesized that TRIF and MyD88 mediate opposing responses to limb ischemia. In this study, we found that IL‐6 levels differ between MyD88 KO and TRIF KO mice, and may be an important determinant of myocyte recovery 1 week after injury. Furthermore, both strains of mice demonstrate an ischemic response after FAL, yet myocyte size and nuclear content was greater in MyD88 KO mice 1 week after injury. This suggests that MyD88 KO mice have a faster recovery after muscle ischemia. Using PCR, we demonstrate that TRIF KO mice contain higher levels of MyD88 mRNA, which may suggest that in the absence of TRIF, MyD88 is upregulated and available to promote inflammation. Additionally, TRIF KO mice had lower levels of the proliferative marker MyoD, suggesting that in the absence of TRIF, regeneration is impaired. This study adds to our previous findings by demonstrating how TRIF and MyD88 may be required to promote inflammation and recovery after limb ischemia.

## Materials and Methods

### Animal models

#### Animals

Male MyD88 KO and TRIF KO mice were used at 10–12 weeks of age and weighed 20–40 g. MyD88 KO and TRIF KO mice were generous gifts from Jay Kolls, MD (Children's Hospital of Pittsburgh, Pittsburgh, PA). Whenever possible, littermates were used as controls for MyD88 KO mice as both strains of mice are raised on antibiotics for 6 weeks. Control C57B6 mice were obtained from Jackson laboratories (Bar Harbor, ME) and were age and weight matched. All procedures conformed to the Guide for the Care and Use of Laboratory Animals published by the United States National Institutes of Health and were in accordance with the policies of the Institutional Animal Use and Care Committee of the University of Pittsburgh (approved – protocol #0911093B‐5).

#### Hindlimb ischemia model

Mice were anesthetized with pentobarbital (0.1 cc/g IP). Bilateral groins were shaved and prepped with iodine solution. Transverse incisions were made in each groin and the femoral structures, were identified. On the right, the external iliac and femoral veins and arteries and all visible branches were ligated with 6‐0 silk as previously described (Messina et al. 2002), avoiding the femoral nerve. On the left, the femoral vessels were exposed but not ligated. Heart rate and respiratory rate were visually monitored to assess depth of anesthesia, and animals were kept warm with a heating lamp. Mice were euthanized by overdose of inhaled isoflurane and cervical dislocation at 4 and 24 h as well as at 1 week after arterial ligation.

#### Laser Doppler perfusion imaging

Excess hairs were removed from the limb with a depilatory cream. The blood flow to both hindlimbs was measured using a Laser Doppler blood flow meter (PERIMED III, Stockholm, Sweden). Three sequential images were obtained and averaged. The entire hindlimb starting from the incision site to the paw was incorporated into the region of interest. Perfusion to each limb was expressed individually, as well as a ratio of ischemic to nonischemic leg.

#### ELISA

Blood was obtained by cardiac puncture. Serum was isolated through centrifugation. IL‐6, TNF*α*, and HMGB1 levels were measured using ELISA kits obtained from R&D Systems (Minneapolis, MN; TNF*α* and IL‐6) and Shino‐test Corporation (Tokyo, Japan; HMGB1).

#### RT‐PCR analysis

Total RNA from ischemic and nonischemic muscles was isolated using RNA STAT‐60 reagent (Amsbio, Lake Forest, CA) following the manufacturer's instructions. Total RNA was then reverse‐transcribed to cDNA using iScript^™^ Reverse Transcription Supermix (Bio‐Rad, Hercules, CA). Amplification reactions for target genes were performed using the GoTaq^®^ Green Master Mix (Promega, Madison, WI) for the same number of cycles for each product. Primers for amplification were: mouse Mac‐1, forward, 5′‐GCCTTGTGTCATGGCTTCAATCTG‐3′, reverse, 5′‐TGATGCTACCGGAGCCATCAATC‐3′; mouse MyoD, forward, 5′‐GCCCGCGCTCCAACTGCTCTGAT‐3′, reverse, 5′‐CCTACGGTGGTGCGCCCTCTGC‐3′; mouse IL‐6, forward, 5′‐TCCAGTTGCCTTCTTGGGACTGAT‐3′, reverse, 5′‐TTGGATGGTCTTGGTCCTTAGCCA‐3′; GAPDH, forward, 5′‐AACCTGCCAAGTATGATGAC‐3′, reverse, 5′‐ATACCAGGAAATGAGCTTGA‐3′. PCR products were separated on the same 1% agarose gel for each gene and identified by ethidium bromide staining. Relative RT‐PCR quantification was performed by expressing target gene signal relative to GAPDH signal using Image J analysis software (Schneider et al. 2012) after color inversion of the image.

#### Histological analysis

Tibialis anterior muscle was collected at sacrifice, fixed in formalin, paraffin embedded, and sectioned (8 *μ*m). This muscle group gives the most consistent response to ischemia induced by femoral artery ligation (Shireman and Quinones 2005; Contreras‐Shannon et al. 2007). Muscle samples obtained 4 and 24 h after ischemia were stained for HMGB1. After washing, sections were incubated with biotinylated goat‐anti‐rat secondary antibody. ABC horseradish peroxidase reagent was added after washing. Antigen detection was performed by adding AEC chromogenic substrate, and sections were counterstained with hematoxylin. Sections were photographed at using a 40× objective and digitally stored. Percent of nuclei staining positive for HMGB1 was quantified using Image J analysis program.

Muscle samples obtained 1 week after ischemia were prepared as described above. Sections were stained with hematoxylin and eosin (H&E) for morphologic evaluation. Three H&E sections 60 *μ*m apart were digitally captured using a 20× objective. Regenerating muscle was characterized by round shape and centrally located nuclei (Charge and Rudnicki 2004). Myofiber cross‐sectional area (CSA) was calculated using Image J after calibrating to a micrometer. The number of nuclei present in each myofiber was also quantified in sections in which regeneration was prominent. Paraffin‐embedded sections were deparaffinized and immunostained for endothelial cell content with isolectin. Three to four images were collected from each of three separate tissue sections 60 *μ*m apart using Olympus Provus I microscope attached to a Nikon camera with a 40× objective. Fractional area of isolectin staining was also quantified using Image J. Image analysis was performed in a blinded fashion.

#### Myoblast proliferation and fusion assay

Mouse myoblasts (C2C12; C3H strain; ATCC^®^ CRL‐1772) were maintained in growth media supplemented with 10% FBS. For proliferation experiments, cells were serum depleted for 2 h before experimentation and incubated with either buffer or IL‐6 (20 ng/mL) for 24 h in the presence of tritiated thymidine (^3^HTdR). The dose of IL‐6 was determined based on preliminary dose–response experiments. Cells were then washed and incubated with 5% trichloroacetic acid overnight, and lysed with 0.3 mol/L NaOH. Incorporation of ^3^HTdR using a scintillation counter and was performed in triplicate for each condition. For the fusion assays, six‐well plates were coated in 50 ng/mL laminin before seeding myoblasts. Myoblasts were then grown to 70–80% confluence in DMEM supplemented with 10% FBS. Growth medium was supplanted with differentiation medium (DMEM plus 2% horse serum) for 4 days. Cells were then stained with DAPI and imaged in four quadrants at 20×. Myoblast fusion index was determined as described (Rando and Blau 1994) by obtaining the ratio of nuclei within fused cells (≥3 nuclei/cell) to total number of nuclei. Experiments were repeated three times.

#### Statistical analysis

Analysis of variance was used to compare multiple means, whereas *t*‐test was used to evaluate differences between two means. *P*s < 0.05 were considered statistically significant. Data are presented as means and standard error of the mean (SEM).

## Results

### Femoral artery ligation results in similar levels of early ischemia and perfusion recovery in control, TRIF KO, and MyD88 KO mice

**Figure 1.**
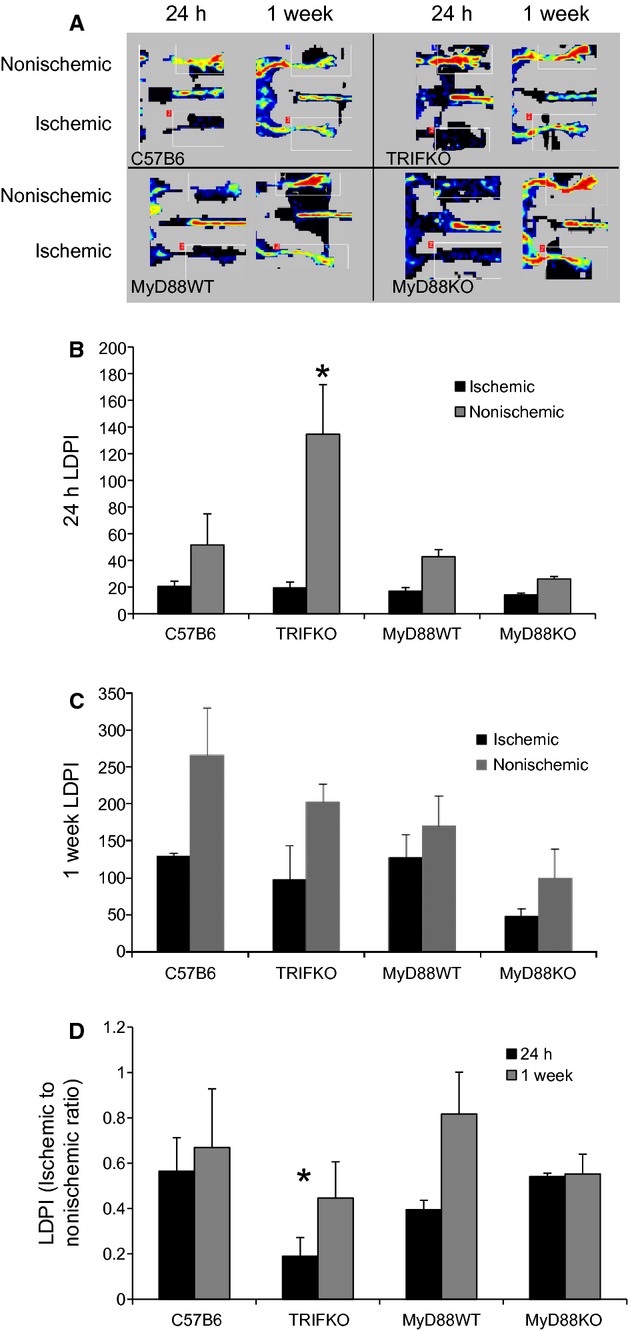
(A) Laser Doppler perfusion images (LDPI) demonstrate the ischemic and nonischemic limbs 24 h and 1 week after right femoral artery ligation. (B) LDPI quantification for each limb after 24 h and 1 week (C). In TRIF KO mice, perfusion to the nonischemic limb is significantly higher than in the other mice (*P =**0.02, ANOVA;* N *=**4/group). (D) LDPI ratios of ischemic to nonischemic limb quantified for 24 h and 1 week (*P =**0.04, ANOVA;* N *=**4/group).

All strains of mice developed similar degrees of ischemia 24 h after FAL. Similarly all strains of mice demonstrated recovery of perfusion to a fraction of what was seen in the nonischemic limb. Both absolute (Fig. 1A–C) and ratios of the ischemic to nonischemic limb (Fig. 1D) are shown. Of note, while the absolute values of perfusion among the strains at 24 h were similar, TRIF KO mice had higher perfusion in the nonischemic hindlimb at the 24 h time point. Thus, the ischemic to nonischemic limb perfusion ratio was lower in that strain (Fig. 1B–D).

### Following FAL, TRIF, and MyD88 KO mice demonstrate temporal differences in serum IL‐6, but not TNFα or HMGB1

**Figure 2.**
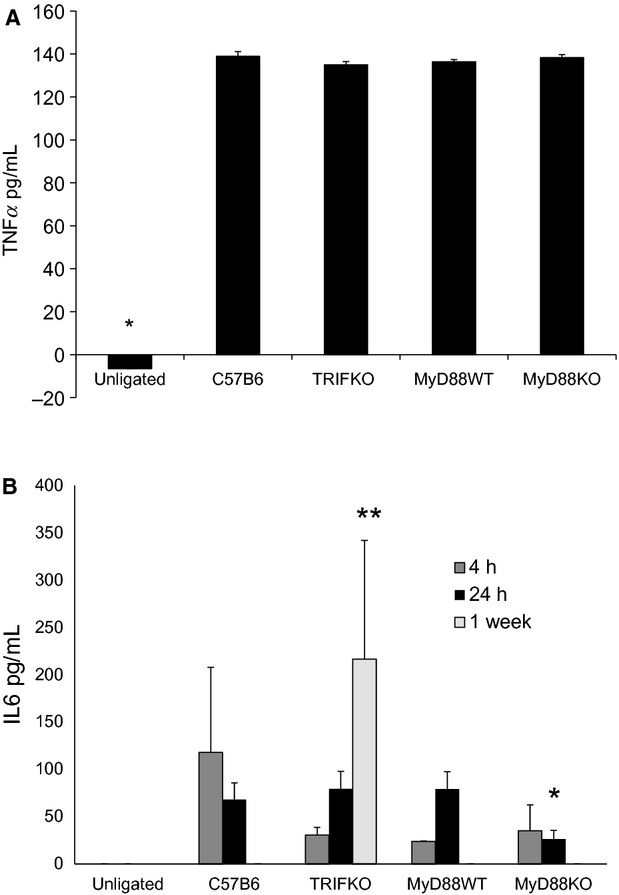
(A) ELISA for TNF*α* was performed from serum 24 h the initiation of femoral artery ligation. There is significant elevation in serum TNF*α* in all surgically manipulated groups compared to unligated animals (*P <**0.001, ANOVA;* N *=**4/group). (B) ELISA for IL‐6 was performed 4 h, 24 h, and 1 week after femoral artery ligation. All mice had significantly elevated IL‐6 levels 4 h after injury when compared to unligated animals (*P <**0.05, ANOVA;* N *=**4–7/group). Twenty‐four hours after injury, MyD88 KO mice demonstrated significantly less IL‐6 in serum than their littermate controls (**P <**0.05, *t*‐test; *N *=**4/group). Only TRIF KO mice had detectable IL‐6 in serum (***P =**0.002, ANOVA,* N *=**4–7/group) 1 week after injury.

We have previously demonstrated that a single dose of neutralizing antibody to HMGB1 administered an hour prior to FAL increased skeletal muscle necrosis 2 weeks later (Sachdev et al. 2013). This suggests that interference with the early signaling events after ischemia leads to profound changes in the recovery process. Thus, we measured the release of inflammatory cytokines as well as HMGB1 at 4 and 24 h after FAL. While TNF*α* release was undetectable at 4 h (not shown), serum levels were elevated in all strains of mice undergoing FAL by the 24 h time point (Fig. 2A). There was no significant difference in TNF*α* levels between the strains. In contrast, FAL induced IL‐6 production which was detectable in all strains of mice at 4 h and remained elevated at 24 h. In MyD88 KO mice, however, IL‐6 concentrations were lower than in control mice at 24 h (Fig. 2B). We also measured both TNF*α* and IL‐6 concentrations 1 week after injury because it has been established that IL‐6 can have both inhibitory and proliferative effects on regenerating myoctes (Haddad et al. 2005; McKay et al. 2009). TNF*α* was undetectable at 1 week (not shown). As IL‐6 is typically elevated at earlier time points after injury, it was not surprising to see IL‐6 levels had returned to baseline and was undetectable in control C57B6, MyD88 WT, and MyD88 KO mice after 1 week. TRIF KO mice were the only strain demonstrating elevated IL‐6 1 week after injury (Fig. 2B).

**Figure 3.**
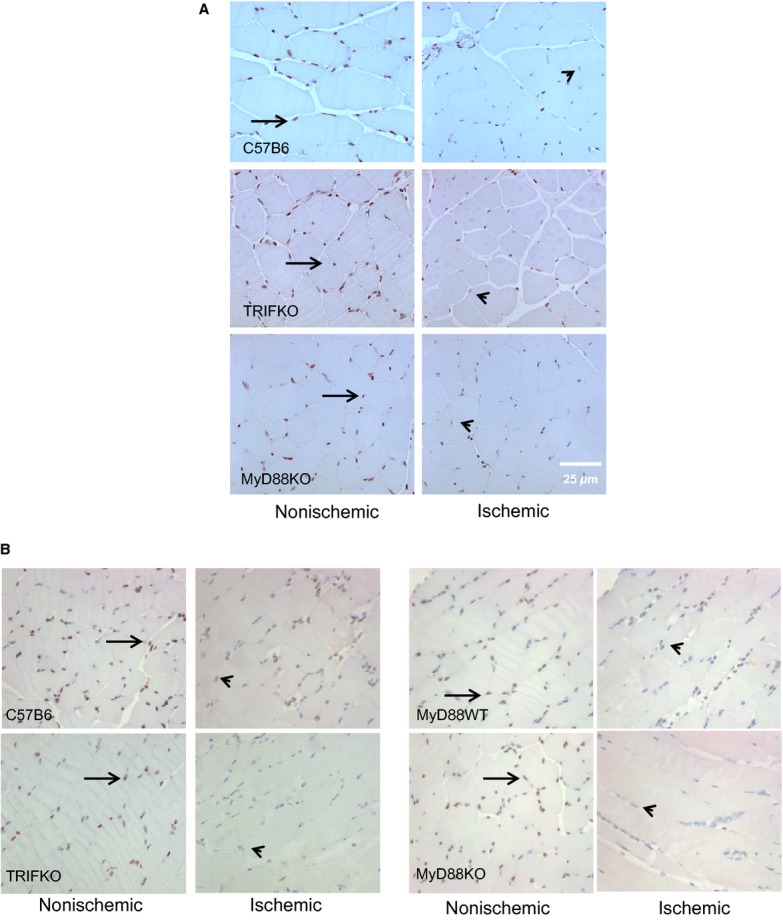
Immunohistochemistry evaluating HMGB1 staining (brown, arrow) in myocyte nuclei (blue, arrowhead) from nonischemic and ischemic limbs 4 (A) and 24 h (B) after femoral artery ligation. The muscle samples were taken from the tibialis anterior compartment.

**Figure 4.**
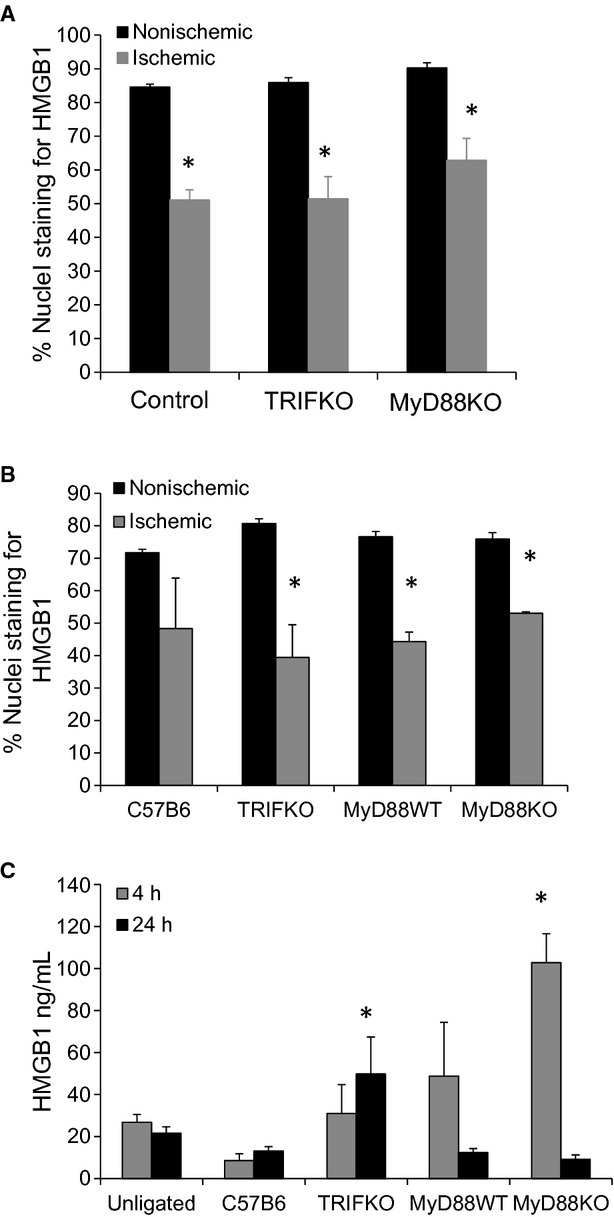
(A) Nuclear staining of HMGB1 was quantified as percent/total nuclear in control, TRIF KO and MyD88 KO mice 4 h after femoral artery ligation (*P <**0.01, *t*‐test ischemic to nonsichemic; *N *=**3–4/group). (B) The same groups including MyD88 WT mice were evaluated for nuclear staining of HMGB1 24 h after femoral artery ligation (*P <**0.01, *t*‐test ischemic to nonsichemic; *N *=**3–4/group). The muscle samples were taken from the tibialis anterior compartment. (C) Serum HMGB1 was determined 4 and 24 h after femoral artery ligation using ELISA (*P <**0.05, ANOVA;* N *=**3–4/group).

We have previously reported that HMGB1 is diminished in myocyte nuclei as early as 4 h after the initiation of ischemia (Sachdev et al. 2013). In other systems, HMGB1 has been shown to have cytokine‐like effects and can be both regenerative as well as damaging. Thus, we measured HMGB1 expression in myocyte nuclei 4 and 24 h after FAL in control, MyD88 KO, and TRIF KO mice as a marker of ischemic sensing. Images were taken in areas of muscle without high levels of inflammatory cell infiltrate, which was determined by preliminary H&E staining from parallel sections. In ischemic muscle, the number of nuclei staining positive for HMGB1 was reduced compared to those from nonischemic hindlimbs across all the strains at both time points (Fig. 3). These differences were significant in all groups of mice at both 4 and 24 h with one exception; while the percent of nuclei staining positive for HMGB1 was less in ischemic limbs of control C57B6 mice at 24 h, the difference was not significant (Fig. 4A and B). Interestingly, a corresponding increase in serum HMGB1, as measured by ELISA, was only seen in MyD88 KO and TRIF KO mice. Serum HMGB1 levels remained unchanged in control mice undergoing FAL in comparison to untreated mice (Fig. 4C).

### TRIF is required for myoblast but not inflammatory cell infiltration into ischemic muscle

**Figure 5.**
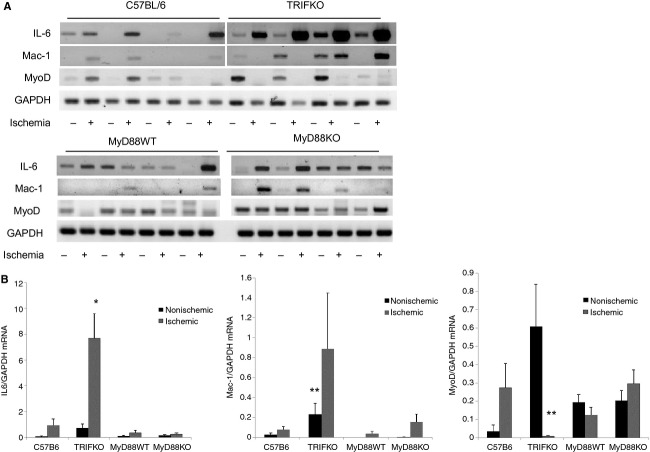
(A) RT‐PCR evaluating mRNA levels of IL‐6, Mac‐1, MyoD, and GAPDH in nonischemic (L) and ischemic (R) tibialis anterior 24 h after femoral artery ligation. (B) Band intensity from RT‐PCR results were quantified using Image J after color inversion of the image. Blots separated by white space were run on the same gel as neighboring blots. Results were normalized to GAPDH and demonstrated for both nonischemic and ischemic limbs (*P <**0.001, ANOVA;* N *=**4/group; **P <**0.05, ANOVA;* N *=**4/group).

Because we determined that there were differences in serum cytokines between MyD88 KO and TRIF KO mice, we investigated inflammatory and proliferative markers in the muscles as well. Inflammatory cytokine expression, leukocyte infiltration and mobilization, and proliferation of stem cells are all elements of the initial injury response to ischemia. We have previously demonstrated that TRIF KO mice exhibited pronounced PMN infiltration 24 h after ischemia in comparison to either control or MyD88 KO mice (Sachdev et al. 2012). To further characterize the inflammatory and myoblastic state of both ischemic and nonischemic muscle, muscle samples were analyzed for the expression of IL‐6, Mac‐1 integrin (a marker of both neutrophils and macrophages), and Myo‐D, a myoblast marker expressed during myogenesis (Lepper et al. 2011), using RT‐PCR (Fig. 5). TRIF KO mice demonstrated significant differences when compared to the other strains. Most notably, IL‐6 was significantly upregulated in the ischemic muscle of TRIF KO as compared to the other strains. Mac‐1 integrin was also elevated in TRIF KO mice in both ischemic and nonischemic limbs. However, MyoD expression was noticeably deficient in the ischemic muscle from TRIF KO mice (Fig. 5).

### One week after FAL, absence of MyD88 signaling is associated with larger myofiber cross‐sectional area and greater nuclear content per myofiber without significant differences in tissue vascularity

**Figure 6.**
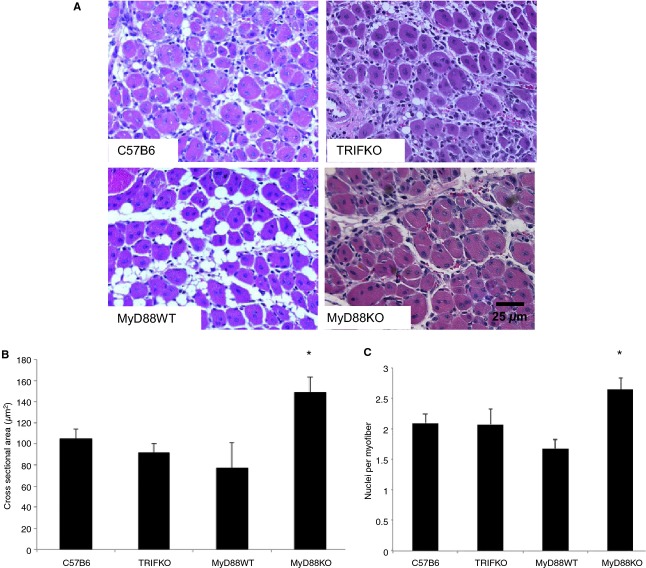
(A) Tibialis anterior from ischemic limbs was obtained after sacrifice 1 week after femoral artery ligation, sectioned, and stained with H&E. Regenerating myocytes are characterized by circular shape and centrally located nuclei. Myocyte cross‐sectional area was determined using Image J analysis from four nonoverlapping images taken from three consecutive sections 8 microns apart with a 20× objective. Average cross‐sectional area (B) and number of nuclei per myofiber (C) is demonstrated for each group (*P<0.05, ANOVA;* N *=**4–5/group).

**Figure 7.**
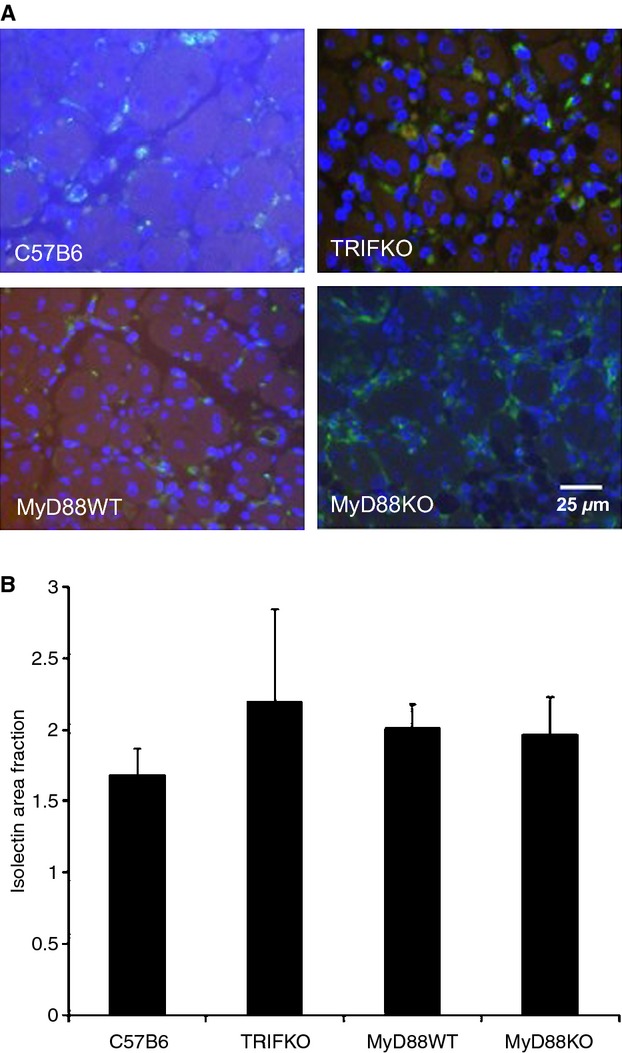
(A) Isolectin staining (green) in ischemic hindlimbs was assessed using a 40× objective 2 weeks after ischemic injury. Contrast was adjusted equally for all images to allow for visualization of green and blue. (B) Area fraction of isolectin staining in each strain is shown, indicating no significant quantitative difference in staining across the strains.

At early (24 h) and later (1 week) time points, IL‐6 levels differed among the strains. In order to assess a potential effect of those cytokine differences, histology was performed on tibialis anterior muscles 1 week after injury. Necrosis, inflammation, and regeneration were prominent features across all the animal groups. However, myofiber CSA was significantly larger in the ischemic hindlimbs of MyD88 KO mice than in other mouse strains tested. Mirroring the size differences, the number of nuclei per myofiber was also significantly higher in MyD88 KO mice, indicative of myoblast differentiation and maturation (Fig. 6). Angiogenesis, as quantified by the number of isolectin‐positive endothelial cells in the ischemic muscle samples, was similar across all the mouse strains (Fig. 7).

### MyD88 is upregulated in muscle tissue from TRIF KO mice

**Figure 8.**
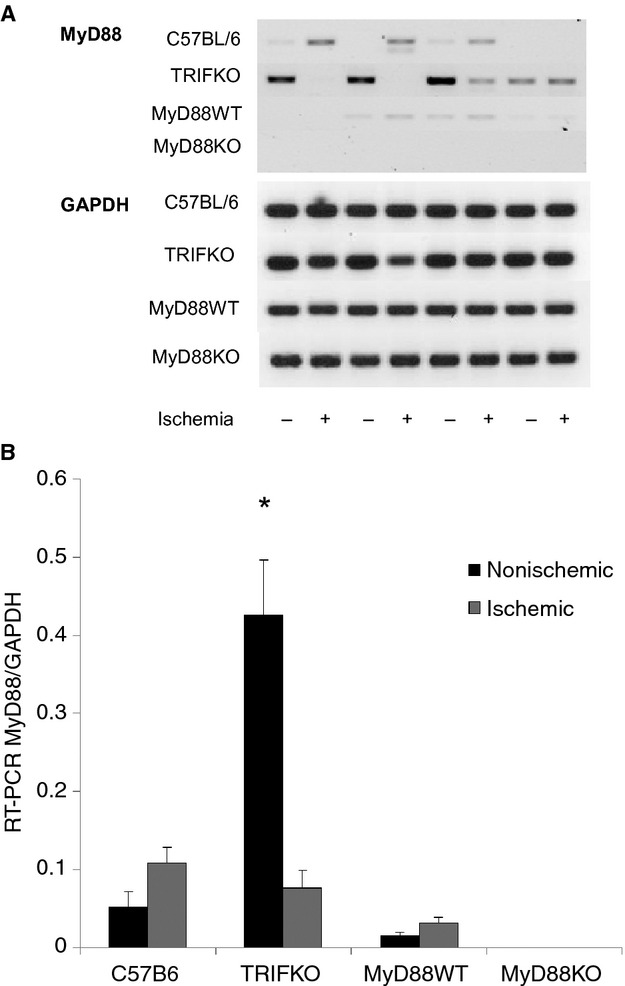
(A) RT‐PCR for MyD88 as well as GAPDH was performed in both nonischemic (L) and ischemic (R) anterior tibialis muscle from control, TRIF KO, MyD88 WT and MyD88 KO mice. (B) Band intensity for MyD88 was normalized to GAPDH after color inversion, and expressed in both nonischemic and ischemic limbs (*P *=**0.004, ANOVA;* N *=**4/group).

One explanation for the differential histologic and cytokine differences between MyD88 KO and TRIF KO mice is that TRIF counteracts the actions of MyD88 as a means of promoting a balanced response to inflammation. Thus, the pronounced damage seen in TRIF KO mice may be due to unopposed MyD88 signaling. To test this hypothesis, MyD88 expression was examined by RT‐PCR in muscle samples collected 24 h after FAL in all animal strains. MyD88 mRNA was significantly increased in the nonischemic muscle from TRIF KO mice but not in the C57B6 mice. No MyD88 mRNA was detected in the MyD88 KO mice, confirming absence of MyD88 in those mice (Fig. 8).

### IL‐6 attenuates myoblast proliferation and fusion in vitro

**Figure 9.**
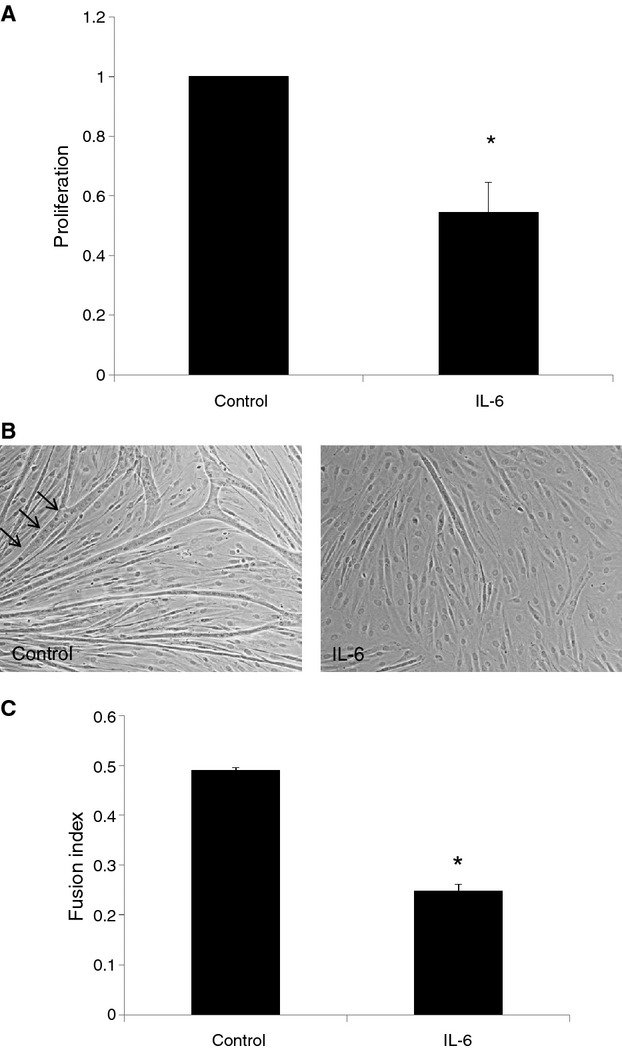
(A) Cultured human myoblasts were treated with control buffer, or IL‐6 20 ng/mL in the presence of ^3H^thymidine. Radioactive uptake was quantitated in triplicate for each group after 24 h. Results demonstrate counts per million of ^3H^thymidine as a measure of proliferation. *P *<**0.01, *t*‐test; *N *=**3. (B and C) Cultured human myoblasts were seeded on laminin‐coated plates and grown to 70–80% confluence before treatment with differentiation serum and either control buffer or IL‐6 (20 ng/mL). After 4 days, cells were stained with DAPI (B) and fusion index was calculated (C). Results demonstrate the average of three separate experiments. *P *<**0.001, *t*‐test; *N *=**3.

In vitro studies were performed to assess the effects of IL‐6 on myoblast proliferation. This was based on the differences in IL‐6 levels observed between the strains, particularly at early time points. IL‐6 diminished myoblast proliferation as compared to cells treated with control buffer (Fig. 9A). Additionally, treatment with IL‐6 significantly inhibited myoblast fusion in response to differentiation serum with fewer multinucleated cells formed (Fig. 9B and C).

## Discussion

Muscle regeneration after injury is a complex process that involves the clearance of damaged myofibers, satellite cell proliferation, myoblast fusion, and incorporation of new myocytes into regenerating muscle (Charge and Rudnicki 2004; Serrano and Muñoz‐Cánoves 2010; Lepper et al. 2011; Relaix and Zammit 2012). Mature muscle fibers are terminally differentiated – thus addition of new genetic material to promote hypertrophy in the setting of muscle loading or repair depends on the incorporation of satellite and myoblast cells within in the muscle (Mitchell and Pavlath 2001; Serrano et al. 2008). Inflammatory pathways play an important role in clearing cellular debris and secreting cytokines that regulate regeneration (Bondesen et al. 2004; Vaquero et al. 2011). We have previously demonstrated that TLR2 and 4, both of which mediate inflammation in response to alarmins, may play complimentary roles in muscle regeneration and angiogenesis after ischemia (Sachdev et al. 2013). Specifically, the absence of TLR2 in C57B6 mice impairs regeneration. Absence of MyD88, an adaptor molecule common in signal transduction for both TLR2 and TLR4, had a dramatic protective effect. There was little evidence of sustained injury in MyD88 KO mice despite perfusion imaging confirming that the hindlimbs had been rendered ischemic 2 weeks earlier (Sachdev et al. 2012). In that study, absence of TRIF, a signaling mediator for TLR4 and TLR3, resulted in prominent necrosis and little evidence of muscle regeneration.

One explanation for these findings is that, in the absence of TLR2, TLR4‐mediated inflammation predominates and impairs regeneration through a dominant pathway. Others have shown that TLR4 mediates upregulation of TLR2 (Li et al. 2009), and our previous data suggest that TLR2 as well as TRIF are protective against extensive necrosis (Sachdev et al. 2012). Thus, the onset of muscle ischemia under normal circumstances may initiate a cascade of events that promotes both inflammation and repair through the activation of TLR4, TLR2, MyD88, and TRIF.

**Figure 10.**
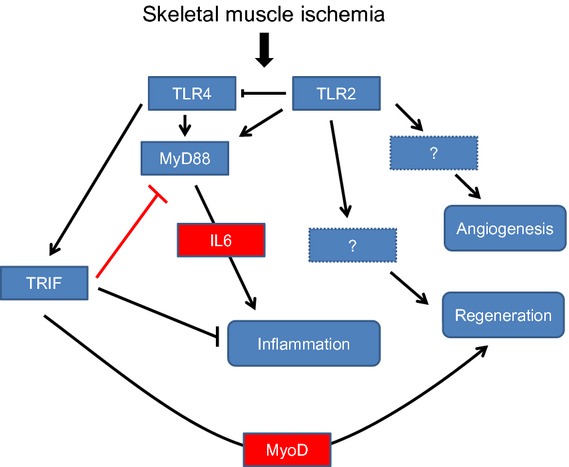
Schematic demonstrating relationship of TLR2 and TLR4 as well as MyD88 and TRIF to muscle inflammation and regeneration after limb ischemia.

Our current study demonstrates that MyD88 KO and TRIF KO mice exhibit important differences in their responses to FAL at both early and late time points, and that MyD88 and TRIF may in part regulate muscle regeneration through IL‐6 (Fig. 10). Twenty‐four hours after muscle ischemia, serum IL‐6 levels were attenuated in the absence of MyD88 signaling. IL‐6 has defined roles in muscle physiology and is released during strenuous exercise (Steensberg et al. 2002; Haddad et al. 2005; Serrano et al. 2008; McKay et al. 2009; Kobara et al. 2010). Its role in regulating muscle regeneration is multifactorial – in some systems, it is proregenerative while in others, the presence of IL‐6 diminishes myoblast proliferation and differentiation (Steensberg et al. 2002; Haddad et al. 2005; Frost et al. 2006; McKay et al. 2009). In our study, MyD88 KO mice had lower levels of IL‐6 early after injury, as well as larger myocytes 1 week after ischemic injury when compared to controls. These data would suggest that one way in which MyD88 KO mice may ultimately have an improved recovery phenotype after FAL is through lower levels of IL‐6 soon after injury. In vitro, we showed that IL‐6 inhibited myoblast proliferation and fusion, also supporting the hypothesis that attenuation of IL‐6 in MyD88 KO mice may result in faster regeneration. In other models of tissue regeneration such as partial hepatectomy, IL‐6 levels were similarly downregulated in the setting of MyD88 deficiency which was ultimately associated with a proregenerative phenotype (Vaquero et al. 2011).

It has been described that IL‐6 may also have a sustaining role in muscle satellite cell proliferation after certain types of exercises such as lengthening (McKay et al. 2009). Thus, the role of IL‐6 is likely to be complex, and the timing of its peaks may be important. In our study, only TRIF KO mice were observed to have persistently elevated serum IL‐6 levels 1 week after FAL, whereas the other strains had undetectable levels. These mice developed relatively smaller regenerating myocytes and had reduced MyoD expression in the ischemic but not the nonischemic limb. Clearly, elevated IL‐6 did not prevent regeneration at 1 week, but may have contributed to lower levels of MyoD‐positive cells in that limb. Of note MyD88 expression was significantly upregulated in the nonischemic limbs of TRIF KO mice. This finding is worth mentioning because FAL in one limb may have global consequences that affect the nonischemic limb as well. Furthermore, the elevated MyD88 expression in the nonischemic limb of TRIF KO mice may suggest that MyD88 expression is higher in TRIF KO mice in response to ischemia. This may result in an increase in the overall inflammatory state.

The nonischemic limbs of the TRIF KO mice exhibited a number of surprising features. Blood flow appeared to be increased in the left hindlimb of TRIF KO mice compared to other animals 24 h after FAL. Both Mac‐1 and MyD88 had significantly higher expression on the nonischemic sides when compared to other strains. Furthermore, while MyoD expression was significantly lower in the ischemic limbs of TRIF KO mice, expression was elevated in the nonischemic limb. These data suggest that the lack of MyoD in TRIF KO mice is not necessarily because of an inherent deficiency of the protein and supports the idea that there may be systemic effects of arterial ligation that affects the unligated limb. Of the cytokines and systemic proteins we measured in the serum after FAL, IL‐6 demonstrated the most significant differences. We also measured serum HMGB1 levels and examined nuclear HMGB1 staining within the muscle tissue itself because we have previously reported that ischemia induces the mobilization and release of nuclear HMGB1 in myocytes (Sachdev et al. 2013). Local and systemic HMGB1 may represent an important signal of tissue damage that can initiate both injurious and regenerative effects through TLR signaling pathways. HMGB1 has been shown to behave as a cytokine that mediates end‐organ damage in shock as well as in hepatic ischemia–reperfusion injury (Tsung et al. 2005; Izuishi et al. 2006; Levy et al. 2007; Mollen et al. 2008). Others have reported that HMGB1 can recruit mesoangioblasts to areas of muscle injury and is proangiogenic (Palumbo et al. 2004; van Beijnum et al. 2006; Mitola et al. 2006; Chavakis et al. 2007; De Mori et al. 2007). Interestingly, while all animals in our current study demonstrated loss of nuclear HMGB1 in the setting of ischemia 4–24 h after FAL, only MyD88 KO mice and TRIF KO mice had detectable serum HMGB1 levels at these time points. One hypothesis is that TLR4, the common upstream receptor of TRIF and MyD88, mediates tissue sequestration of HMGB1 in the ECM after cellular release. Other plasma proteins such as fibrinogen have been found to extravasate from blood vessels in injured muscle and exert their effects by lodging in the surrounding matrix (Serrano and Muñoz‐Cánoves 2010). This is an area of active investigation in our laboratory.

In conclusion, innate immune pathways are critical to the muscle repair process in the setting of injury. Our data support that MyD88 and TRIF mediate opposing responses to skeletal muscle ischemia perhaps working in concert to prevent an exaggerated inflammatory response while promoting regeneration. MyD88 is required for IL‐6 release into the serum, while TRIF appears to be required for myoblast mobilization into ischemic tissue. The role of HMGB1 and TLR4 in mediating the upstream early events after skeletal muscle ischemia remains a focus of further investigation.

## Conflict of Interest

None declared.
